# Supplementary material for: Impact of Physical Contact on Preterm Infants’ Vital Sign Response to Live Music Therapy
Source: Int J Environ Res Public Health. 2022 Aug 3;19(15):9524. doi: 10.3390/ijerph19159524 (PMC9368366; doi:10.3390/ijerph19159524)
Supplement: Supplementary file 1 [file ijerph-19-09524-s001.zip › ijerph-1806763-supplementary.pdf]

Supplementary Table S1. Vital sign values in 40 stable preterm infants before and after 604 music therapy sessions with and without physical contact.

Vital Sign	Type of Measurement	Physical Contact Mean (95% CI) (*n* = 159)	No Physical Contact Mean (95% CI) (*n* = 444)	*p*-Value *
Heart rate (beats/min)	Baseline	158.7 (156.4–160.9)	162.2 (160.9–163.5)	0.007
	After therapy	151.3 (148.6–153.9)	153.2 (151.6–154.7)	0.209
	Difference **	-7.4 (-9.8–(-5.1))	-9.0 (-10.3–(-7.7))	0.232
Respiratory rate (breaths/min)	Baseline	53.5 (51.0–55.9)	57.4 (55.9–59.0)	0.009
	After therapy	41.7 (39.6–43.8)	43.6 (42.1–45.0)	0.169
	Difference **	-11.8 (-14.7–(-8.8))	-13.9 (-15.8–(-11.9))	0.258
SaO_2 (%)	Baseline	95.7 (95.0–96.3)	94.7 (94.3–95.2)	0.027
	After therapy	97.3 (96.8–97.9)	96.9 (96.5–97.2)	0.192
	Difference **	1.7 (1.0–2.3)	2.2 (1.8–2.5)	0.193

* two-sided *t*-test with pooled variance. **between baseline and after therapy.

Supplementary Table S2. Model information of the multivariable analyses.

		Main Analysis				Sensitivity Analysis			
**Vital Sign**	**Parameter**	**Estimate**	**Lower 95% CI**	**Upper 95 % CI**	***p*-Value**	**Estimate**	**Lower 95% CI**	**Upper 95 % CI**	***p*-Value**
Heart rate	Intercept	252.7	249.8	255.4	<.0001	139.2	118.4	160.0	<.0001
	Physical contact	1.3	-1.5	4.1	0.362	1.6	-0.8	4.0	0.194
	No physical contact (reference)	0.0	0.0	0.0		0.0	0.0	0.0	
	Corrected GA	-3.0	-3.1	-3.0	<.0001	-1.9	-2.2	-1.7	<.0001
	Baseline heart rate					0.5	0.4	0.6	<.0001
Respiratory rate	Intercept	86.0	69.8	102.2	<.0001	76.2	60.5	91.9	<.0001
	Physical contact	-1.0	-3.5	1.5	0.444	0.0	-2.5	2.6	0.970
	No physical contact (reference)	0.0	0.0	0.0		0.0	0.0	0.0	
	Corrected GA	-1.3	-1.8	-0.8	<.0001	-1.2	-1.6	-0.8	<.0001
	Baseline respiratory rate					0.1	0.1	0.2	0.001
SaO_2	Intercept	80.7	80.4	81.0	<.0001	54.9	49.7	60.1	<.0001
	Physical contact	-0.1	-0.5	0.3	0.541	-0.3	-0.7	0.1	0.1
	No physical contact (reference)	0.0	0.0	0.0		0.0	0.0	0.0	

	Corrected GA	0.5	0.5	0.5	<.0001	0.4	0.4	0.4	<.0001
	Baseline SaO_2					0.3	0.3	0.4	<.0001

CI = confidence interval, GA = gestational age

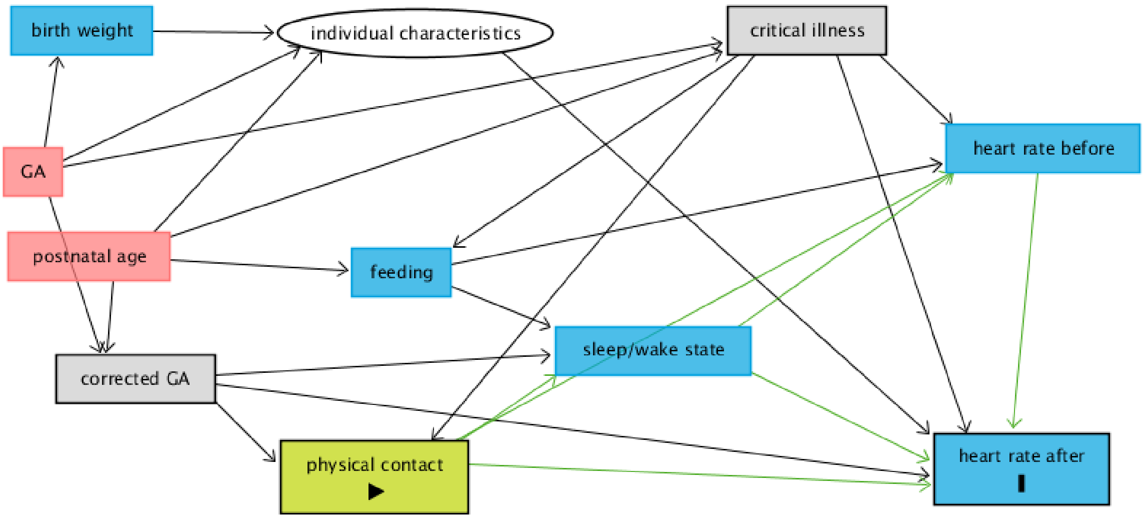

Figure S1. Directed acyclic graph on the total effect of physical contact during music therapy on the heart rate. Green = exposure, heart rate after = outcome, grey = adjusted confunders in the final model, blue = ancestor of outcome, red = ancestor of exposure and the outcome, white circle = unobserved factors. GA = gestational age.

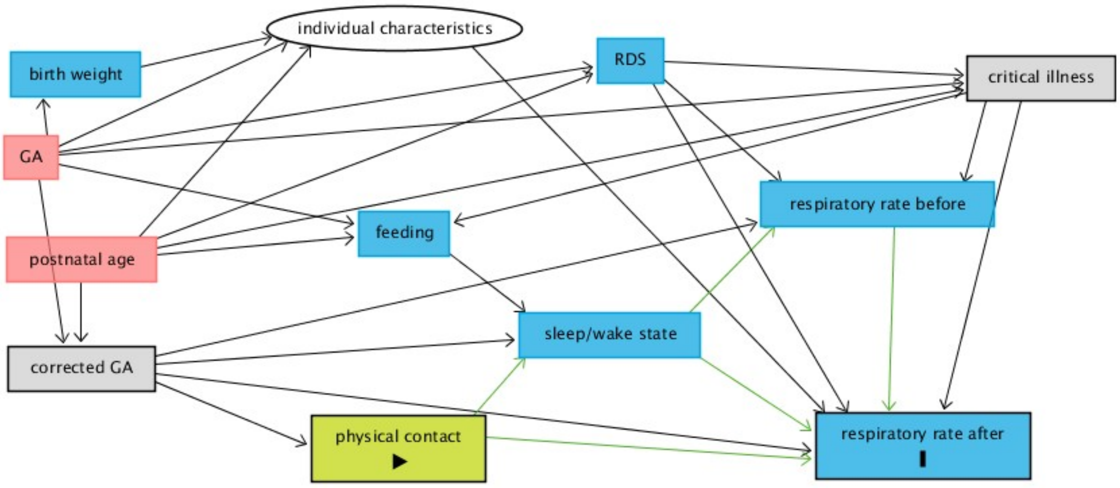

Figure S2. Directed acyclic graph on the total effect of physical contact during music therapy on the respiratory rate. Green = exposure, respiratory rate after = outcome, grey = adjusted confounders in the final model, blue = ancestor of outcome, red = ancestor of exposure and the outcome, white circle = unobserved factors. GA = gestational age, RDS = respiratory distress syndrome.

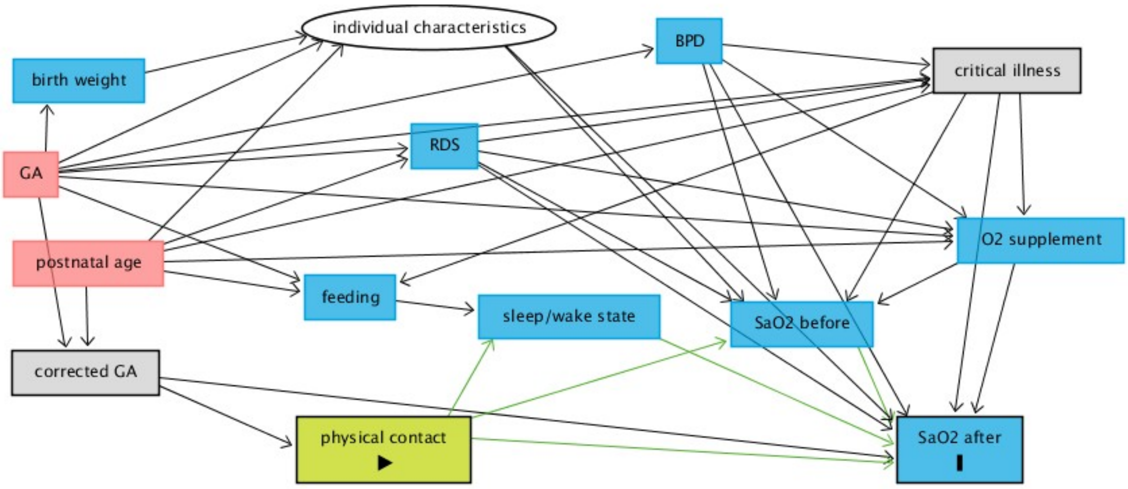

Figure S3. Directed acyclic graph on the total effect of physical contact during music therapy on the oxygen saturation. Green = exposure, SaO2 after = outcome, grey = adjusted confounders in the final model, blue = ancestor of outcome, red = ancestor of exposure and the outcome, white circle = unobserved factors. BPD = bronchopulmonary dysplasia, GA = gestational age, RDS = respiratory distress syndrome, SaO2 = oxygen saturation.
